# Urotensin II Inhibits Skeletal Muscle Glucose Transport Signaling Pathways via the NADPH Oxidase Pathway

**DOI:** 10.1371/journal.pone.0076796

**Published:** 2013-10-08

**Authors:** Hong-Xia Wang, Xin-Rui Wu, Hui Yang, Chun-Lin Yin, Li-Jin Shi, Xue-Jiang Wang

**Affiliations:** 1 Department of Physiology and Pathophysiology, School of Basic Medical Sciences, Capital Medical University, Beijing, China; 2 Department of Cardiology, Xuan Wu Hospital, Capital Medical University, Beijing, China; 3 Department of Neurology, the First Affiliated Hospital of Xinxiang Medical College, Xinxiang, China; University of Texas Health Science Center at Houston, United States of America

## Abstract

Our previous studies have demonstrated that the urotensin (UII) and its receptor are up-regulated in the skeletal muscle of mice with type II diabetes mellitus (T2DM), but the significance of UII in skeletal muscle insulin resistance remains unknown. The purpose of this study was to investigate the effect of UII on NADPH oxidase and glucose transport signaling pathways in the skeletal muscle of mice with T2DM and in C2C12 mouse myotube cells. KK/upj-AY/J mice (KK) mice were divided into the following groups: KK group, with saline treatment for 2 weeks; KK+ urantide group, with daily 30 µg/kg body weight injections over the same time period of urantide, a potent urotensin II antagonist peptide; Non-diabetic C57BL/6J mice were used as normal controls. After urantide treatment, mice were subjected to an intraperitoneal glucose tolerance test, in addition to measurements of the levels of ROS, NADPH oxidase and the phosphorylated AKT, PKC and ERK. C2C12 cells were incubated with serum-free DMEM for 24 hours before conducting the experiments, and then administrated with 100 nM UII for 2 hours or 24 hours. Urantide treatment improved glucose tolerance, decreased the translocation of the NADPH subunits p40-phox and p47-phox, and increased levels of the phosphorylated PKC, AKT and ERK. In contrast, UII treatment increased ROS production and p47-phox and p67-phox translocation, and decreased the phosphorylated AKT, ERK1/2 and p38MAPK; Apocynin abrogated this effect. In conclusion, UII increased ROS production by NADPH oxidase, leading to the inhibition of signaling pathways involving glucose transport, such as AKT/PKC/ERK. Our data imply a role for UII at the molecular level in glucose homeostasis, and possibly in skeletal muscle insulin resistance in T2DM.

## Introduction

Urotensin II (UII) is a vasoactive peptide that was first discovered in teleost fishes, and later in mammals and humans [1,2]. UII acts by binding to the G protein coupled receptor GPR14 (now known as UT) [3], and have been detected in cardiac and vascular tissues, and the spinal cord, central nervous system, kidney, liver and pancreas [4]. Importantly, UII and UT are abundant in the skeletal muscle of mouse and monkey, and radio-ligand binding assay has shown that UT binds [^125^I]UII with high affinity in skeletal muscle [5]. Besides its important role in the cardiovascular system, UII also participates in metabolic regulation and plays a significant role in diabetes and its complications [6,7]. Our previous studies demonstrated that the UII/UT system is up-regulated in the skeletal muscle of mice with type II diabetes mellitus (T2DM), and UII inhibited insulin-stimulated 2-DG uptake in skeletal muscle [8]. We speculated that skeletal muscle-derived UII might be involved as an autocrine/paracrine factor in the pathogenesis of skeletal muscle insulin resistance (IR), although the mechanism remains unclear.

IR, the major defect of T2DM, is a common pathophysiological state in which higher than normal concentrations of insulin are required to exert its biological effect in target tissues such as the skeletal muscle, adipose tissue and liver [9]. Considering the skeletal muscle accounts for the majority of insulin-mediated glucose disposal in the post-prandial state, skeletal muscle IR contributes significantly to the metabolic derangements seen in T2DM patients. The precise molecular mechanisms responsible for insulin resistance remain incompletely understood, however, particularly in skeletal muscle. Emerging data indicated that oxidative stress due to increased reactive oxygen species (ROS) generation and/or compromised antioxidant systems represents an important factor in the progression of insulin resistance [10]. One of the main sources of ROS is NADPH oxidase (NOX), a multi-protein enzyme complex that uses NADPH as a substrate to convert molecular oxygen to ROS. Components of NADPH oxidase complex of phagocytes include the membrane-bound cytochrome b558, composed of 2 subunits, p22-phox and gp91-phox, and 4 cytosolic subunits, p47-phox, p67-phox, p40-phox, and the small GTP-binding protein, Rac. Moreover, expression of gp91phox, p22phox, p40 phox, p47phox, and p67phox have been documented in skeletal muscle [11]. Wei et al found NADPH oxidase activation and ROS generation play an important role in Ang II-induced inhibition of insulin signaling in skeletal muscle cells [12]. Given these data, studies are warranted to ascertain whether UII mediates skeletal muscle IR by increasing ROS production via NADPH oxidase.

In the present study, we sought to determine whether UII antagonism improved glucose tolerance by decreasing the oxidative state in KK mice, and to investigate the effect of UII on ROS production and on glucose transport signaling in C2C12 mouse myotube cells. We study the effects of UII on ROS production and NADPH oxidase levels, and its involvement in the regulation of the AKT/PKC/ERK signaling pathway.

## Results

### Urantide improves glucose tolerance in KK mice

**Figure 1:**
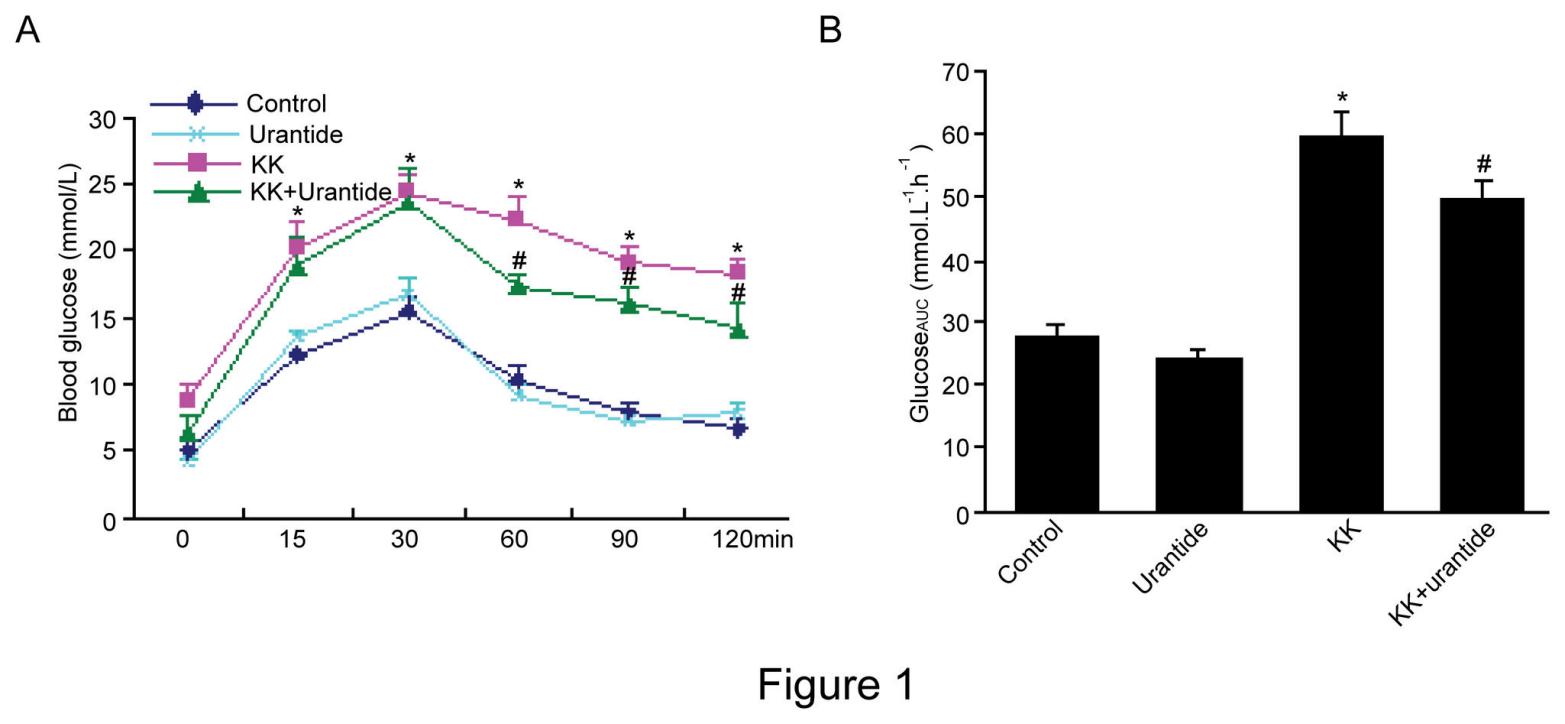
Effect of urantide on blood glucose levels and area under the curve (AUC) in the glucose tolerance test. KK mice were administrated urantide (30 µg/kg/day) for 14 days prior to calculation of glucose loading (A) and area under the curve (AUC) of changes in blood glucose levels (B). Control: C57BL/6J mice were treated with saline for 14 days; Urantide: C57BL/6J mice were treated with urantide for 14 days; KK:KK mice were treated with saline for 14 days; KK+Urantide: KK mice were treated with urantide for 14 days. Each value is mean±SD (n=6-8 for each group, *P<0.05 vs control; ^#^ *P*<0.05 vs KK group).

Based on the result of the intraperitoneal glucose tolerance test (IGTT), the KK group mice remained hyperglycemic and glucose intolerant 2 weeks after saline treatment. In contrast, blood glucose was reduced in the KK+Urantide group compared to the KK group (p <0.05). Glycemic excursion was normal in the C57BL/6J control group and Urantide group (Figure 1).

### Urantide decreases MDA content and increases the anti-oxidative effect in KK mice

**Figure 2:**
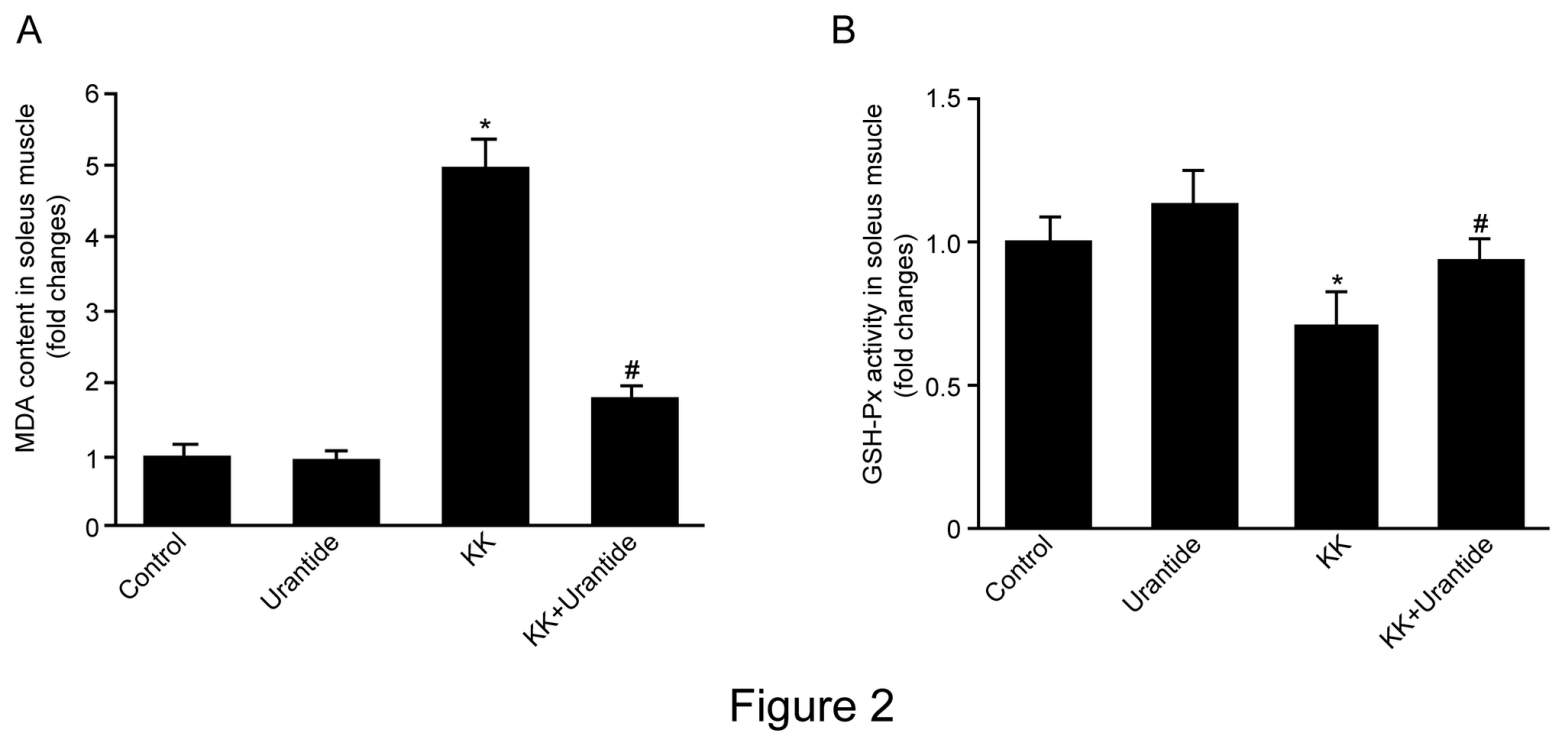
Effect of urantide on MDA content and GSH-Px activity in soleus muscle in mice. KK mice were administrated urantide (30 µg/kg/day) for 14 days prior to measurement of (**A**) MDA content by thiobarbituric acid assay and (**B**) GSH-Px activity using a Glutathione Assay Kit. Control (C57BL mice were treated with saline for 14 days), Urantide (C57BL mice were treated with urantide for 14 days), KK (KK mice were treated with saline for 14 days), KK+Urantide (KK mice were treated with urantide for 14 days). Control: C57BL/6J mice were treated with saline for 14 days; Urantide: C57BL/6J mice were treated with urantide for 14 days; KK:KK mice were treated with saline for 14 days; KK+Urantide: KK mice were treated with urantide for 14 days. Data are presented as the relative increase compared with control (100%; n=6-8, *P<0.05 vs control; ^#^ *P*<0.05 vs KK group).

Malondialdehyde (MDA), a product of membrane lipid peroxidation, levels in soleus tissue were significantly higher in the KK group than in the control group. Importantly, MDA levels were significantly lower in the KK+Urantide group than in the KK group (Figure 2A).

GSH-Px activity in soleus muscle tissue was significantly lower in the KK group than in the control group. In contrast, GSH-Px activity was significantly higher in the KK+Urantide group than in the KK group (Figure 2B).

### Urantide decreases the translocation of p40-phox and p67-phox protein in skeletal muscle in KK mice

**Figure 3:**
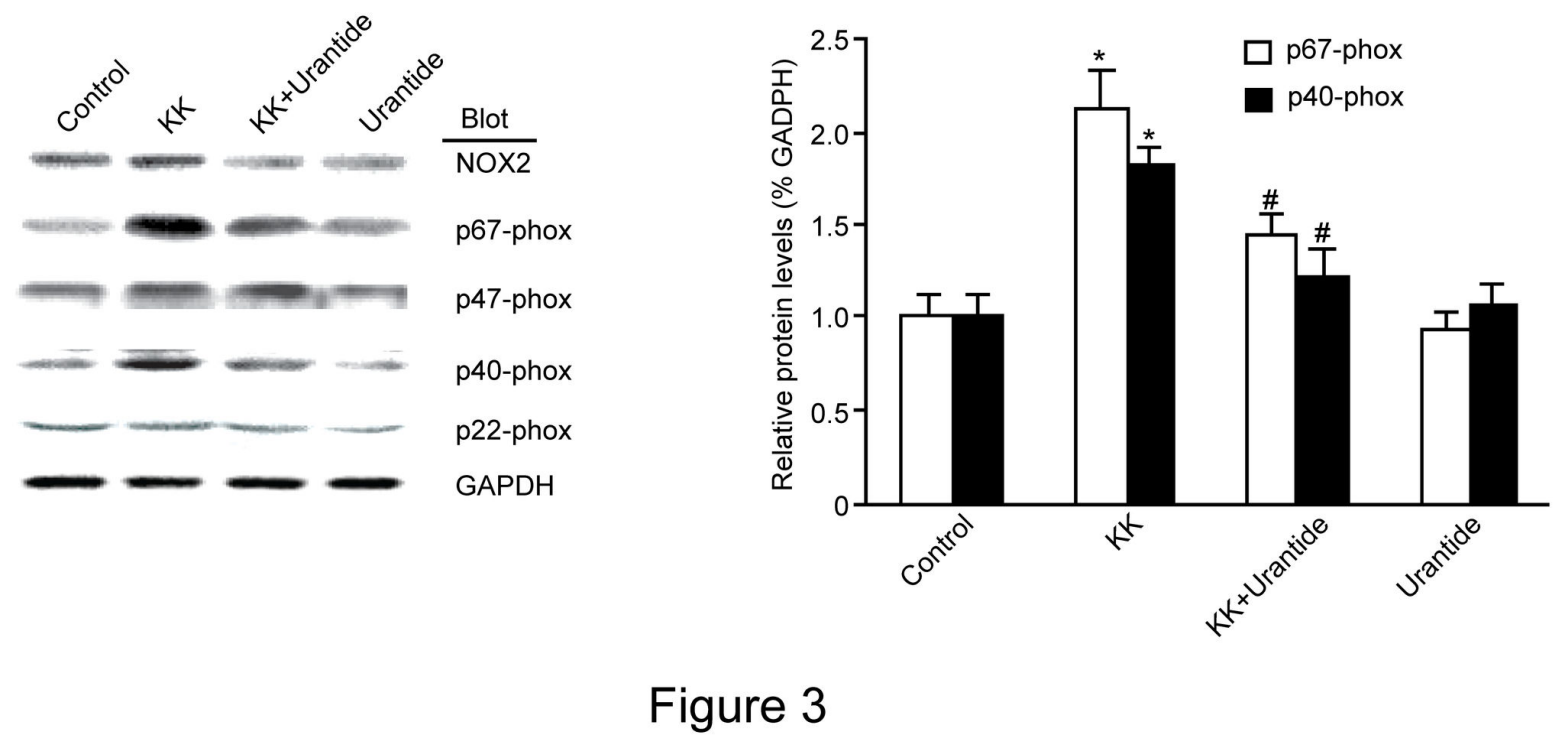
Effect of urantide on NADPH oxidase subunit expression in soleus muscle in mice. KK mice were administrated urantide (30 µg/kg/day) for 14 days prior to western blot analysis with antibodies against NOX2, p67-phox, p47-phox, p40^phox^ and p22^phox^. Equal loading was confirmed by reprobing the membranes with GAPDH antibody. Control: C57BL/6J mice were treated with saline for 14 days; Urantide: C57BL/6J mice were treated with urantide for 14 days; KK:KK mice were treated with saline for 14 days; KK+Urantide: KK mice were treated with urantide for 14 days. Data are presented as the relative increase compared with control (100%; n=3, *P<0.05 vs control; ^#^ *P*<0.05 vs KK group).

The NADPH oxidase complex, including gp91-phox (NOX2), p67-phox, p47-phox, p40^phox^ and p22^phox^ (NOX4), is expressed in skeletal muscle and regulates the production of H_2_O_2_. We next measured the translocation of NOX2, p67-phox, p47-phox, p40^phox^ and p22^phox^ in soleus muscle using western blot. As shown in Figure 3, the translocation of p67-phox and p40-phox was significantly higher in the KK group than in the control group. Conversely, urantide treatment significantly decreased p67-phox and p40-phox protein translocation. NOX2 and p47-phox were not affected.

### Urantide increases GLUT4 translocation and the phosphorylated PKC, AKT and ERK in skeletal muscle in KK mice

**Figure 4:**
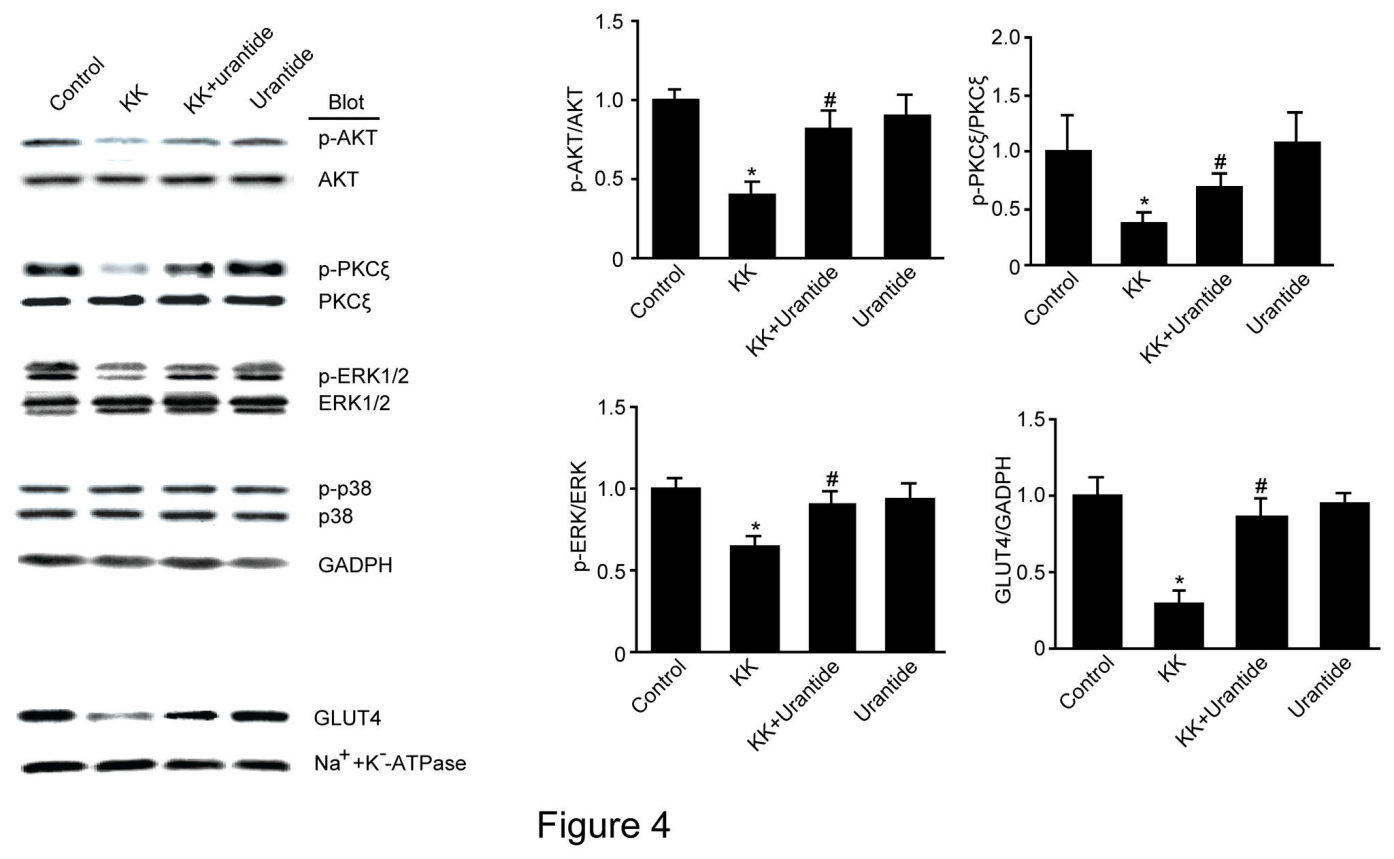
Effect of urantide on GLUT4 protein expression and PKC, AKT, ERK protein phosphorylation. KK mice were administrated urantide (30 µg/kg/day) for 14 days prior to western blot analysis with antibodies against GLUT4, PKC, AKT, ERK and p38MAPK. Equal loading was confirmed by reprobing the membranes with GAPDH or Na^+^-K^+^-ATPase antibody. Control: C57BL/6J mice were treated with saline for 14 days; Urantide: C57BL/6J mice were treated with urantide for 14 days; KK:KK mice were treated with saline for 14 days; KK+Urantide: KK mice were treated with urantide for 14 days. Data are presented as the relative increase compared with control (100%; n=3, *P<0.05 vs control; ^#^ *P*<0.05 vs KK group).

We next investigated GLUT4 translocation, the predominant glucose transporter isoform in skeletal muscle. Compared to the control group, GLUT4 translocation was significantly decreased in the KK group, in contrast, urantide significantly increased GLUT4 translocation (Figure 4). We next investigated the phosphorylation status of protein kinase B (PKB)/Akt and protein kinase Cζ (PKCζ), which are well-known metabolic switches, and the phosphorylation status of extracellular signal-regulated kinase (ERK)1/2 MAPK and p38 MAPK, which are known mediators in glucose transport. Compared to the control group, PKC, AKT and ERK protein phosphorylation were significantly lower in the KK group than in the control group, and urantide significantly increased PKC, AKT and ERK protein phosphorylation.

### UII elevates ROS levels and decreases SOD and GSH-Px activity in C2C12 cells

**Figure 5:**
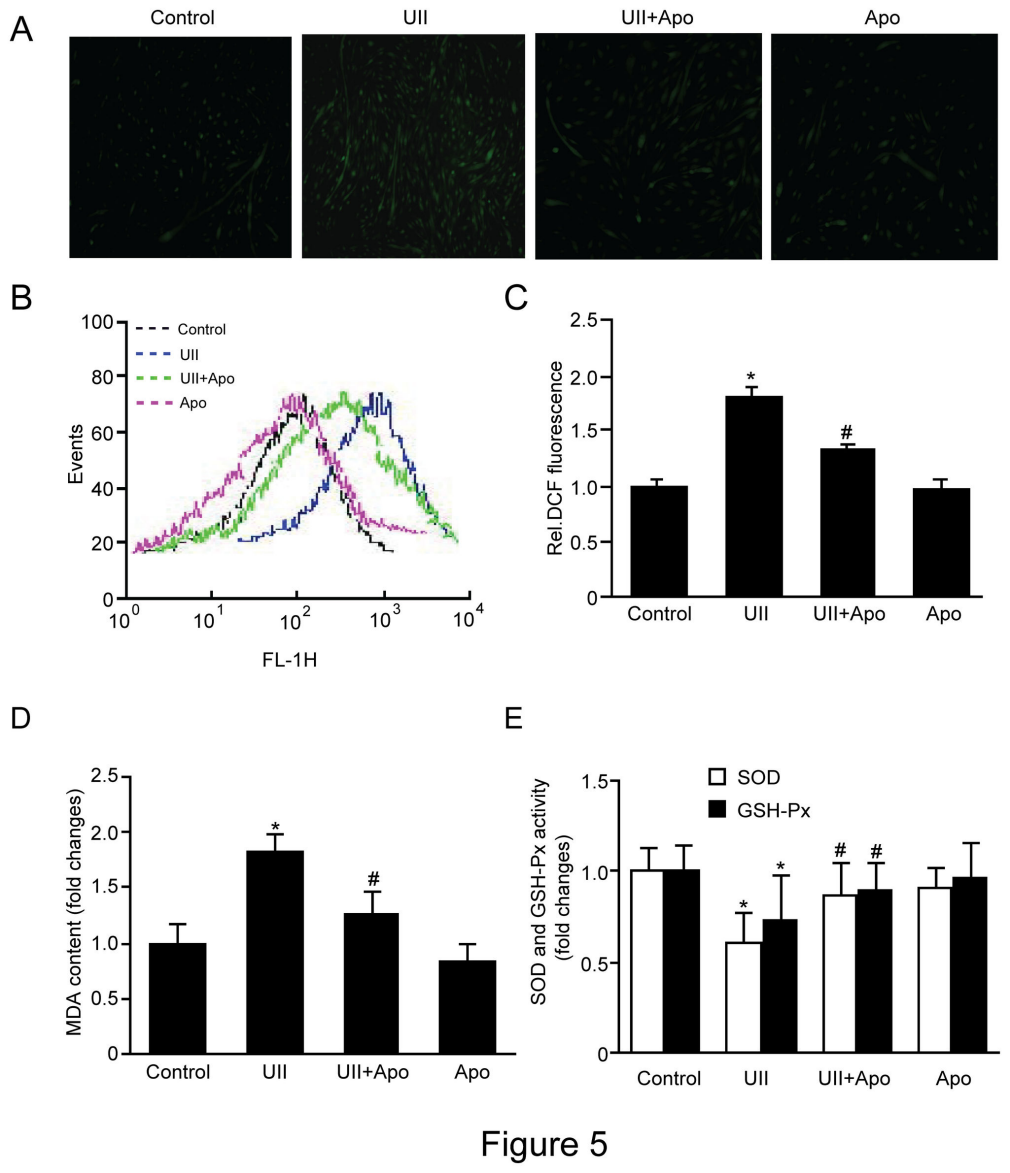
Effect of UII on ROS production, MDA content and antioxidase activity. C2C12 cells were stimulated with 100 nM UII for 2h, and apocynin was administrated for 30 minutes before stimulation with UII. (**A**) ROS levels were visualized by DCF staining (n=3). (**B**) ROS levels were assessed by DCF fluorescence using flow cytometry (n=3). (**C**) ROS levels were evaluated by DCF fluorescence in a microplate reader. (**D**) MDA content was measured by the thiobarbituric acid assay. (**E**) SOD and GSH-Px activity were measured using a Glutathione Assay Kit. Control: cells were incubated with fresh FCS free DMEM for 24h; Apo: cells were incubated with fresh FCS free DMEM containing 200 µmol/L apocynin for 24h; UII: cells were treated with UII (100 nmol/L) for 24h; UII+Apo: cells were treated with apocynin (200 µmol/L) for 30min, and then incubated with UII for 24h. Data are presented as the relative increase compared with control (100%; n=6-8, *P<0.05 vs control group; ^#^ *P*<0.05 vs UII group).

To investigate whether UII can increase ROS levels, C2C12 cells were exposed to 100 nM UII for 2 h and stained with DCF. A strong increase in fluorescence in stimulated C2C12 compared to controls indicated increased ROS levels (Figure 5A). Alternatively, C2C12 cells were treated with UII for 2 h, and ROS levels were measured by DCF fluorescence in a flow cytometer (Figure 5B) and in a microplate reader (Figure 5C).

Pretreatment with the ROS inhibitor apocynin (200 µmol/L) for 30 min significantly decreased UII induced ROS elevation. Moreover, UII treatment increased MDA content (Figure 5D) and decreased SOD and GSH-Px activity (Figure 5E).

### UII elevates p47-phox and p67-phox protein translocation in C2C12 cells

**Figure 6:**
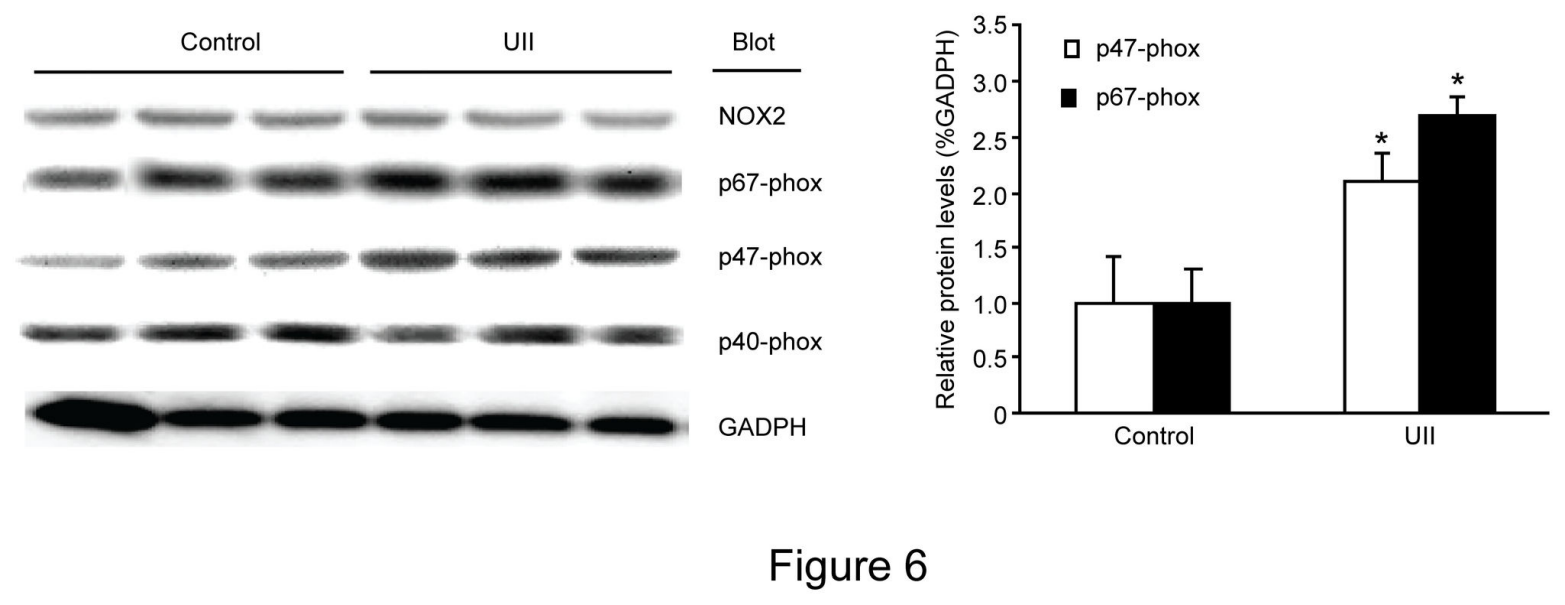
Effect of UII on NADPH oxidase subunit expression. C2C12 cells were stimulated with 100 nM UII for 2h after treatment with apocynin for 30 minutes. Western blot analysis was performed with antibodies against NOX2, p67-phox, p47-phox, p40^phox^ and p22^phox^. Equal loading was confirmed by reprobing the membranes with GAPDH antibody. Control: cells were incubated with fresh FCS free DMEM for 24h; UII: cells were treated with UII (100 nmol/L) for 24h. Data are presented as the relative increase compared with control (100%; n=3, *P<0.05 vs group; ^#^ *P*<0.05 vs UII group).

We evaluated the role of NADPH oxidase in response to UII. C2C12 was stimulated with 100 nM UII, and the translocation of the NADPH oxidase subunits NOX2, p67-phox, p47-phox, p40^phox^ and p22^phox^ was analyzed by western blot (Figure 6). Treatment with UII resulted in robust up-regulation of p47-phox and p67-phox protein translocation.

### UII decreases the phosphorylation status of kinases involved in the glucose transport signaling in C2C12 cells

**Figure 7:**
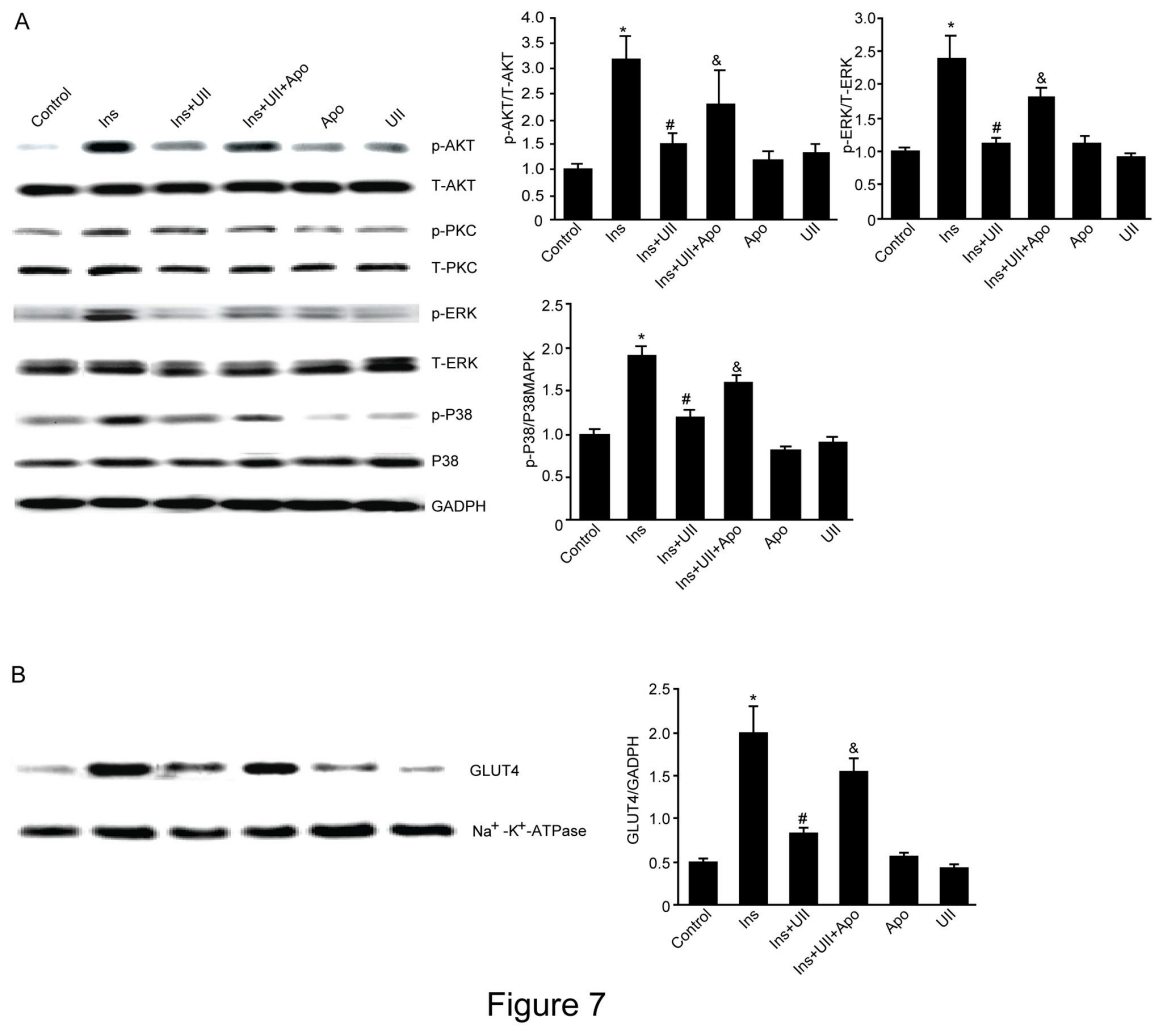
Effect of UII on GLUT4 protein expression and PKC, AKT, ERK protein phosphorylation. C2C12 was stimulated with 10 nM insulin or 100 nM UII for 20 minutes after administration of apocynin for 30 minutes. Western blot analysis was performed with antibodies against GLUT4 from membrane fractions, and PKC, AKT, ERK, p38MAPK from whole cell lysates. Equal loading was confirmed by reprobing the membranes with Na^+^-k^+^-ATPase or GAPDH antibody. Control: cells were incubated with fresh FCS free DMEM for 24h; Apo: cells were incubated with fresh FCS free DMEM containing 200 µmol/L apocynin for 24h; UII: cells were treated with UII (100 nmol/L) for 24h; UII+Apo: cells were treated with apocynin (200 µmol/L) for 30min, and then incubated with UII for 24h. Data are presented as the relative increase compared with control (100%; n=3, *P<0.05 vs control; ^#^ *P*<0.05 vs Ins group; & *P*<0.05 vs Ins+UII group).

To determine the signaling pathways targeted by UII, C2C12 cells were treated with 100 nM UII and the phosphorylation status of AKT, ERK1/2 and p38MAPK and GLUT4 translocation were analyzed by Western blot. UII decreased insulin-stimulated GLUT4 translocation, AKT, ERK and p38 phosphorylation, but UII alone had no this effect. Pretreatment with apocynin for 30 min abrogated the UII-induced GLUT4 translocation and the phosphorylated forms of AKT, ERK and P38MAPK in C2C12 cells (Figure 7).

## Discussion

Insulin resistance in peripheral tissues is an essential element in the pathogenesis of T2DM. The KK mouse, an animal model of T2DM, is characterized by insulin resistance, hyperglycemia and hyperinsulinemia [13]. In the present study, we demonstrated for the first time that urantide administration improves glucose tolerance in this mouse model. Furthermore, urantide treatment decreased the level of MDA, as well as p67-phox, p40-phox translocation, and increased the phospho-form of AKT, PKC and ERK in the skeletal muscle of the KK mouse. These results indicated that urantide may improve skeletal muscle glucose transport signaling pathways by decreasing ROS production via NADPH oxidase. In order to further study the effect of UII on ROS production and the signaling pathways involved in glucose transport, we evaluated the effect of UII on ROS production, NADPH oxidases subunits translocations, and AKT/PKC/ERK/P38MAPK phosphorylation in C2C12 cells. Pretreatment with 100 nM UII increased ROS production, NADPH oxidases subunits translocations, and decreased AKT/PKC/ERK phosphorylation. Taken together, these data suggest that UII can impair skeletal muscle glucose transport signaling pathways by increasing ROS production, and that urantide can ameliorate these effects.

ROS plays an important role as signaling molecules in skeletal muscle IR, and NADPH oxidases are known to be involved in the ROS production in skeletal muscle [14,15]. NADPH oxidases consist of five subunits, including p67-phox, p47-phox, p40-phox, p22-phox and gp91-phox (NOX2) [16]. Isabel et al found that UII failed to induce angiogenesis in NOX2 knockdown and in NOX2 knockout mice, and that UII increased NOX2 transcription in endothelial cells [17]. Moreover, it has been shown that UII significantly increases ROS levels in pulmonary artery smooth muscle cells, and this response was accompanied by elevated protein levels of the NADPH oxidases subunits p22-phox and NOX4 [18]. In addition, Chen et al showed that UII increased ROS production in cardiac fibroblasts [19]. Our results demonstrated that UII significantly increased ROS levels in C2C12 cells, and this effect was accompanied by elevated protein translocation of the NADPH oxidase subunits p47-phox and p67-phox. These findings demonstrate for the first time that UII increases ROS production and NADPH oxidases subunits translocations in C2C12 cells.

Glucose transport is mediated by insulin receptor substrate-1 (IRS-1), phosphatidylinositol 3-kinase(PI3-kinase), and other signaling molecules, and down stream substrates activated by PI-3-kinase include protein kinase B (PKB)/Akt and protein kinase C-ζ (PKC-ζ) [20]. Treatment of C2C12 cells with UII significantly decreased levels of GLUT4 translocation and phospho-AKT activated by insulin, and apocynin reversed these effects. We also determined that UII decreased phosphorylation of ERK1/2 and p38 MAPK, which are known mediators of glucose transport [21], and that apocynin improved these effects. However, treatment of C2C12 cells with UII had no effect on phospho-AKT, ERK and p38 MAPK levels, maybe because C2C12 cell is different from vascular smooth muscle cell. Collectively, these results demonstrated that UII impaired skeletal muscle glucose transport signaling pathways partly via a mechanism involving ROS production.

In this study, we found some effects of urantide in vivo are different from those of UII in vitro. For example, p-PKC was increased by urantide in vivo but it was not changed by UII in vitro, however, p38MAPK was not changed in vivo, whereas it was decreased by UII. A close relationship between chronic inflammation and skeletal muscle insulin resistance had been established [10], so the reasons for these differences may be due to the role of inflammatory cytokine. Thus it should further discuss if these proteins are also phosphorylated by inflammatory cytokines.

## Conclusions

In the present study, we have shown for the first time that UII stimulates NADPH oxidases subunits translocation, enhances the levels of ROS and inhibits phosphorylation of AKT, PKC, ERK1/2 and p38 MAPK. Our results suggest that UII, as an autocrine/paracrine factor derived from skeletal muscle, affects glucose homeostasis by increasing ROS production, and may be involved in the incidence and development of diabetes.

## Materials and Methods

### Animals, chemicals, reagents and antibodies

All procedures were approved by and performed in accordance with the Animal Care and Use Committee of Capital Medical University (20100610). All animals received humane care, and the experimental protocol was approved by the Committee of Laboratory Animals according to institutional guidelines.

Male diabetic KK/upj-AY/J, C57BL/6J mice (8 weeks old) and C2C12 cells were purchased from the Chinese Academy of Medical Sciences, Peking Union Medical College Institute of Laboratory Animal Science. KK/upj-AY/J mice were originally generated by repeated cross-breeding of yellow obese mice with KK mice. Nakamura disclosed the characteristics of diabetes mellitus in an inbred strain of mice named KK [22]. The yellow obese mice bear a dominant gene of Ay, which shows yellow coat color in heterozygous form. The yellow Ay mutation produces a mouse that also presents hyperinsulinemia, insulin resistance, leptin resistance, hyperglycemia, and hyperleptinemia. The cross-breed of both strains, the KKAy mouse, is a model of obesity, hypertriglyceridemia, and hypercholesteremia and also shows severely elevated plasma glucose and insulin levels, and is therefore diabetic and insulin resistant [23]. The mice were housed in air-conditioned, specific pathogen-free animal quarters with lighting from 8:00 to 21:00, and the mice were fed a standard chow diet, and food and water were provided ad libitum.

KK mice were divided into two groups receiving either saline or 30 µg/kg body weight of urantide for 2 weeks. Then the mice were killed under isoflurane anesthesia, and the soleus muscles were collected.

All chemicals were purchased from Sigma Chemical (St Louis, MO, USA). Dulbecco’s modified Eagle medium (DMEM), fetal calf serum and other culture products were purchased from Gibco BRL (Life Technologies, Paisley, UK). UII was form Phoenix Pharmaceuticals (Belmont, CA). Urantide was purchased from Peptides (Louisville, KY, USA). Anti-AKT, anti-phospho-AKT (Thr308), anti-PKC,anti-phospho-PKC (Thr 410/403), anti-ERK1/2 and anti-phospho-ERK1/2 (Thr202/Tyr204) were obtained from Cell Signaling (Beverly, MA, USA). Anti-gp91-phox, anti-p67-phox, anti-p47-phox, anti-p40-phox, anti-p22-phox and GLUT4 were obtained from Santa Cruz Biotechnology.

### Chronic infusion of the UII receptor antagonist urantide in KK mice

KK mice were anesthetized by an intraperitoneal injection of a mixture of ketamine and xylazine (9:1, 2 ml/kg). Before implantation, a mini-osmotic pump was filled with urantide solution and kept in autoclaved saline (37°C) for 8 h. After a transverse incision in the scapular region, the osmotic pump was implanted subcutaneously between the scapula and urantide was infused for 14 days at a dose of 30 µg/kg/day. Sham-operated mice were anesthetized and operated upon using the same procedure, except for the implantation of the osmotic pump.

### Cell culture and treatment

Monolayers of C2C12 myoblasts were maintained at subconfluent conditions in growth media containing DMEM with 4.5 g/L glucose, 1% streptomycin/penicillin and 20% fetal bovine serum. Near-confluent cells (~80% confluence) were differentiated by lowering the serum concentration to 2% calf serum. Cells were maintained for 3–7 days to obtain myotubes and grown in a humidified, 37°C incubator with ambient oxygen and 5% CO_2_. C2C12 cells were randomly divided into 4 groups. The Control group was incubated with fresh FCS free DMEM for 24h. The UII group was treated with UII (100 nmol/L) for 24h [8]. The UII+Apo group was treated with apocynin (200 µmol/L) for 30min, and then incubated with UII for 24h. The Apo group was incubated with fresh FCS free DMEM containing 200 µmol/L apocynin for 24h.

### Intraperitoneal glucose tolerance test (IGTT)

All mice in the study underwent an IGTT after a 6h fast. Each mouse was treated by intraperitoneal gavage with 2 g/kg body weight of glucose diluted in distilled water (120 mg/ml). Blood samples from the tail vein were collected at 0 (before glucose injection), 30, 60 and 120 min after glucose challenge. The blood glucose concentration was determined using an Accu-Chek Advantage glucometer (Roche, Germany).

### Determination of ROS, T-SOD, GSH-PX and malondialdehyde (MDA) activities

The dye 2’, 7’-dichlorofluorescein diacetate (DCF-DA, Molecular Probes, Cat NO: E004, Nanjing Jiancheng Biotechnology Institute, China) was used to detect changes in cellular ROS levels. Briefly, C2C12 cells were incorporated with DCF-DA for 30 min and washed with PBS three times. Fluorescent images in 24 well plates were immediately visualized with a microscope (Olympus DX-63, Japan). ROS levels were determined in 96 well plates by measuring the fluorescence strength with a BioTek Synergy 2 microplate reader (Winooski, VT, USA). The intensity of the fluorescence was expressed as arbitrary units per microgram of proteins. The intensity of fluorescence in 6 wells plate was analyzed by flow cytometry.

Soleus muscle tissue homogenates were obtained from frozen tissue in cold physiologic saline. Homogenates were then centrifuged at 3,000 g for 15 min at 4°C, and the protein concentrations of the supernatants were determined using a BCA Protein Assay Kit (BioTeke Corporation, China). The total activities of SOD (T-SOD) and GSH-Px, as well as the malondialdehyde (MDA) levels, were determined using colorimetric assays with commercial kits (Cat NO: A001-1, A005, A003-1, Nanjing Jiancheng Biotechnology Institute, China). The T-SOD activity was measured using a colorimetric assay at 560 nm, and the results are presented as U per milligram of protein in the tissue. The GSH-Px activity was determined as a function of the rate of GSH consumption in a reaction in which GSH is converted to GSSG at the same time as H_2_O_2_ is reduced to H_2_O. MDA levels were determined using a spectrophotometer at 532 nm to monitor the reaction with 2-thiobarbituric acid (TBA). The results are presented as nmol per milligram of tissue protein.

### Immunoblotting

Protein samples were prepared from C2C12 cells and soleus muscle tissue, and western blot analysis was performed as described [24]. In total, 50µg protein was loaded on SDS-PAGE gels, transferred to PVDF membrane and incubated with primary antibodies Anti-AKT (1:1000), anti-phospho-AKT (1:800), anti-PKC (1:1000), anti-phospho-PKC (1:800), anti-ERK1/2(1:1000), anti-phospho-ERK1/2 (1:800), anti-gp91-phox (1:500), anti-p67-phox (1:500), anti-p47-phox (1:500), anti-p40-phox (1:500), anti-p22-phox (1:500) or GLUT4 (1:200). Blots were developed by use of a chemiluminescent system, and densitometry analysis was carried out using a Gel-pro 4.5 Analyzer (Media Cybernetics).

### NADPH oxidase and GLUT4 translocation

The plasma Membrance-enriched fractions isolated by differential centrifugation and further centrifuged at 100,000×g. Pellets containing the plasma membrane (PM) fractions were resuspended in homogenization buffer and analyzed by Western blot analysis using antibodies to NADPH oxidase and GLUT4. We have verified PM enriched fractions by noting robust Na^+^-K^+^-ATPase α-subunit expression by Western blot and by confirming negligible citrate synthase (mitochondria activities) (data not shown).

### Statistical analysis

All values are expressed as the mean ± SD and were analyzed using SPSS 11.5 (SPSS Inc., Chicago, IL, USA). Differences between groups were assessed by one-way ANOVA or two-way ANOVA, and Bonferroni’s test was used for multiple comparisons. A p-value of < 0.05 was considered significant.
